# PET Imaging of Lung Inflammation with [^18^F]FEDAC, a Radioligand for Translocator Protein (18 kDa)

**DOI:** 10.1371/journal.pone.0045065

**Published:** 2012-09-12

**Authors:** Akiko Hatori, Joji Yui, Tomoteru Yamasaki, Lin Xie, Katsushi Kumata, Masayuki Fujinaga, Yuichiro Yoshida, Masanao Ogawa, Nobuki Nengaki, Kazunori Kawamura, Toshimitsu Fukumura, Ming-Rong Zhang

**Affiliations:** Department of Molecular Probes, Molecular Imaging Center, National Institute of Radiological Sciences, Chiba, Japan; University of Texas, M.D. Anderson Cancer Center, United States of America

## Abstract

**Purpose:**

The translocator protein (18 kDa) (TSPO) is highly expressed on the bronchial and bronchiole epithelium, submucosal glands in intrapulmonary bronchi, pneumocytes and alveolar macrophages in human lung. This study aimed to perform positron emission tomography (PET) imaging of lung inflammation with [^18^F]FEDAC, a specific TSPO radioligand, and to determine cellular sources enriching TSPO expression in the lung.

**Methods:**

An acute lung injury model was prepared by intratracheal administration of lipopolysaccharide (LPS) to rat. Uptake of radioactivity in the rat lungs was measured with small-animal PET after injection of [^18^F]FEDAC. Presence of TSPO was examined in the lung tissue using Western blot and immunohistochemical assays.

**Results:**

The uptake of [^18^F]FEDAC increased in the lung with the progress of inflammation by treatment with LPS. Pretreatment with a TSPO-selective ligand PK11195 showed a significant decrease in the lung uptake of [^18^F]FEDAC due to competitive binding to TSPO. TSPO expression was elevated in the inflamed lung section and its level responded to the [^18^F]FEDAC uptake and severity of inflammation. Increase of TSPO expression was mainly found in the neutrophils and macrophages of inflamed lungs.

**Conclusion:**

From this study we conclude that PET with [^18^F]FEDAC may be a useful tool for imaging TSPO expression and evaluating progress of lung inflammation. Study on human lung using [^18^F]FEDAC-PET is promising.

## Introduction

Acute or chronic lung diseases afflict millions of people around the world each year [1]. It is not easy to elucidate the in vivo pathogenic processes involved in the progress of lung inflammatory disease, such as acute lung injury, acute respiratory distress syndrome, and chronic obstructive pulmonary disease (COPD), which has hampered development and application of suitable therapies and evaluation of therapeutic effects [2]–[4]. Although chest radiographs visualize changes in lung density, this technique does not provide information about disease activity. High-resolution CT shows lung architecture but does not evaluate the activation of inflammatory cells. Lung biopsy is invasive and difficult to repeat, and does not represent the total pulmonary inflammatory burden. Therefore, the development of a noninvasive and reproducible technique would be useful for assessing the pathogenic processes of lung inflammation.

Positron emission tomography (PET) is a molecular and functional imaging modality, which permits repeated and noninvasive determination and quantification of specific biological and pharmacological processes [5], [6]. This technique allows the whole lung to be visualized in vivo. PET imaging with [^18^F]FDG has been used to assess metabolic activity of neutrophils in lung and to elucidate the relationship between migration and activation of neutrophils in pneumonia and bronchiectasis [7]–[10].

PET imaging with [^11^C](*R*)-PK11195, a specific radioligand for translocator protein (18 kDa) (TSPO, formerly known as the peripheral-type benzodiazepine receptor, PBR), has been used to assess activation of macrophages in inflammatory animal models and COPD patients [11]–[13]. TSPO has broad functions related to the regulation of cholesterol transport, synthesis of steroid hormones, porphyrin transport and heme synthesis, apoptosis, cell proliferation, anion transport, regulation of mitochondrial functions, immunomodulations, and inflammation [14], [15]. TSPO is highly expressed on the bronchial and bronchiole epithelium in the lungs of humans and guinea pigs, and on submucosal glands in intrapulmonary bronchi [16], pneumocytes and alveolar macrophages [17] in human, although the pharmacological functions of TSPO in these tissues have not been elucidated clearly.

The aim of this study was to apply a new TSPO radioligand *N*-benzyl-*N*-methyl-2-[7,8-dihydro-7-(2-[^18^F]fluoroethyl)-8-oxo-2-phenyl-9*H*-purin-9-yl]acetamide ([^18^F]FEDAC) [18]–[20] to visualize lung inflammation with PET. An acute lung injury model was prepared by administration of lipopolysaccharide (LPS) to induce response in the rat lung involving a number of inflammatory cells [21]–[26]. Uptake of [^18^F]FEDAC was measured in lung with small-animal PET at several time points after LPS inducement. Presence of TSPO was examined in the lung tissue at the cellular level using Western blot and immunohistochemical assays. Therefore, this study presents the data describing the relationship among the uptake of [^18^F]FEDAC, TSPO expression, and neutrophil and macrophage activation in lung tissue.

## Materials and Methods

### Production of [^18^F]FEDAC and [^11^C](*R*)-PK11195

Radioisotopes ^18^F and ^11^C were produced in-house using a CYPRIS HM-18 cyclotron (Sumitomo Heavy Industry, Tokyo, Japan). [^18^F]FEDAC with >98% radiochemical purity and 145–210 GBq/µmol specific activity was prepared by reacting [^18^F]fluoroethyl bromide with a precursor [18]. [^11^C](*R*)-PK11195 with >99% radiochemical purity and 37–74 GBq/µmol specific activity was prepared by reacting [^11^C]methyl iodide with a desmethyl precursor [27].

### Study Animals

Male Sprague-Dawley rats (6–7 weeks old) were purchased from Japan SLC (Shizuoka, Japan). The rats were housed under a 12-h dark-light cycle and were allowed free access to food pellets and water. The animal experiments were approved by the Animal Ethics Committee of the National Institute of Radiological Sciences (Chiba, Japan) and were carried out according to the recommendations of the Committee for the Care and Use of Laboratory Animals, National Institute of Radiological Sciences.

### Preparation of Rat Model of Acute Lung Injury

About 60 rats were anesthetized with 5% (v/v) isoflurane and then intratracheally administered with 5 mg/kg lipopolysaccharide (*Escherichia coli* 055:B5; Sigma, St. Louis, MO, USA) dissolved in 0.2 mL physiological saline solution, or with 0.05 mL saline only. A 22-gauge catheter was inserted into the trachea through the mouth to directly deliver the LPS solution to the trachea with the aid of a surgical microscope system (Leica M300, Wetzlar, Germany). From these, 44 rats were selected and used for PET imaging experiments of 11 groups (*n* = 4).

### PET Study on Rats

PET scans were performed using a small-animal Inveon PET scanner (Siemens, Knoxville, TN, USA), which provides 159 transaxial slices with 0.796 mm (centre-to-centre) spacing, a 10 cm transaxial field of view (FOV), and a 12.7 cm axial FOV. Before scanning, the rats were anesthetized with 5% (v/v) isoflurane, and maintained thereafter by 1–2% (v/v) isoflurane. Emission scans were acquired for 30 min in 3-dimensional (3D) list mode with an energy window of 350–750 keV, immediately after intravenous injection of [^18^F]FEDAC (8–18 MBq/200 µL, 43–97 pmol). To determine the in vivo specific binding to TSPO, unlabelled PK11195 (1.0 mg, 2.8 µmol/kg) dissolved in 300 µL saline containing 10% ethanol and 5% polysorbate 80 was injected 1 min before injection of [^18^F]FEDAC. For comparison, PET with [^11^C](*R*)-PK11195 (16–19 MBq/200 µL, 0.22–0.26 nmol) was performed on the rats used as controls, LPS-24 h inducement and inhibitory experiment with PK11195. After the PET scans were finished, all rats were sacrificed and radioactivity concentrations in the blood and lungs were measured.

All list-mode acquisition data were sorted into 3D sinograms, which were then Fourier rebinned into 2D sinograms (frames×min: 2×0.5, 3×1, 8×2, 2×5). Dynamic images were reconstructed with filtered back-projection using a Hanning’s filter, and a Nyquist cutoff of 0.5 cycles/pixel. A region of interest was placed on the lung using ASIPro VM (Analysis Tools and System Setup/Diagnostics Tool, Siemens Medical Solutions USA). Regional uptake of radioactivity was decay-corrected to the injection time and was expressed as the standardized uptake value (SUV), which was normalized to the injected radioactivity and body weight. SUV = (radioactivity per millilitre tissue/injected radioactivity) ×gram body weight. Values of areas under the time-activity curves of lung (AUC_0–30__min_, SUV×min) were calculated from 0 min to 30 min after injection.

### Western Blot Assay for TSPO

Parts of the left lobes of rat lungs (*n* = 4) were homogenized in radioimmunoassay precipitation buffer and centrifuged at 2,000 g for 15 min at 4°C. Afterwards, protein concentration in the supernatant was measured using Lowry’s method. The protein samples (60 µg) were separated by SDS-polyacrylamide gel electrophoresis and transferred to polyvinylidene difluoride membranes. After blockade of nonspecific binding, the membranes were incubated with rabbit polyclonal primary antibody against mouse TSPO (NP155, 1∶1000) [28] for 1 h at room temperature, followed by incubation with anti-rabbit horseradish peroxidase-conjugated secondary antibody (1∶5000) for 1 h at room temperature.

Immunoreactive proteins were visualized with the use of enhanced chemiluminescence detection (GE Healthcare, Buckinghamshire, UK). The membranes were reprobed with monoclonal antibody anti-beta-actin (R&D Systems, Minneapolis, MN, USA) diluted 1∶5000 to verify the amount of protein loading in each lane. The ratio of band intensity between TSPO and beta-actin was calculated to correct any variation in the protein gel loading.

### Immunohistochemical Staining Assay

The left upper lobes of rat lungs (*n* = 4) were fixed in 10% buffered formalin and embedded in paraffin. Representative 4-µm-thick sections were selected for hematoxylin-and-eosin (H&E) by use of standard technique and for immunofluorescence staining. For the immunofluorescence staining, nonspecific binding sites on the sections were blocked. The sections were incubated with primary antibody overnight at 4°C. Staining with primary antibody was performed using either a rabbit anti-mouse TSPO (NP155, 1∶1000) [28] or a polyclonal rabbit anti-human myeloperoxidase antibody for detection of neutrophils (1∶50; Abcam, Cambridge, MA, USA). After the first immunoreaction, the sections were incubated with fluorophore (Alexa Fluor 488 or 546 nm)-conjugated secondary antibody for 1 h at room temperature, or without fluorophore followed by the method of tyramide signal amplification (TSA) using the Fluorescein System (PerkinElmer, Waltham, MA, USA). The sections were mounted with a DAPI-containing mounting medium (Vector Laboratories, Burlingame, CA, USA).

Double staining was carried out for TSPO and either macrophages with a monoclonal mouse anti-rat ED1 antibody (1∶100; AbD Serotec, Raleigh, NC, USA), or neutrophils with chloroacetate esterase, a specific marker for neutrophils (Naphthol AS-D chloroacetate esterase kit, Sigma). In the case of chloroacetate esterase staining, counterstaining with hematoxylin was performed for the nuclear staining. To further characterize the inflammatory infiltrate in the lung sections, we examined at least 10 high power fields (×400, 0.035 mm^2^) in each histological section and counted the numbers of TSPO-positive neutrophils and macrophages.

### Statistics

Data were expressed as mean ± SEM. Comparisons were made using the one-way analysis of variance followed by Bonferroni’s post hoc test. The analysis was performed using GraphPad Prism 5 software (GraphPad Software, La Jolla, CA, USA). Differences between groups were considered significant when the p-value was less than 0.05.

## Results

### PET Study

**Figure 1:**
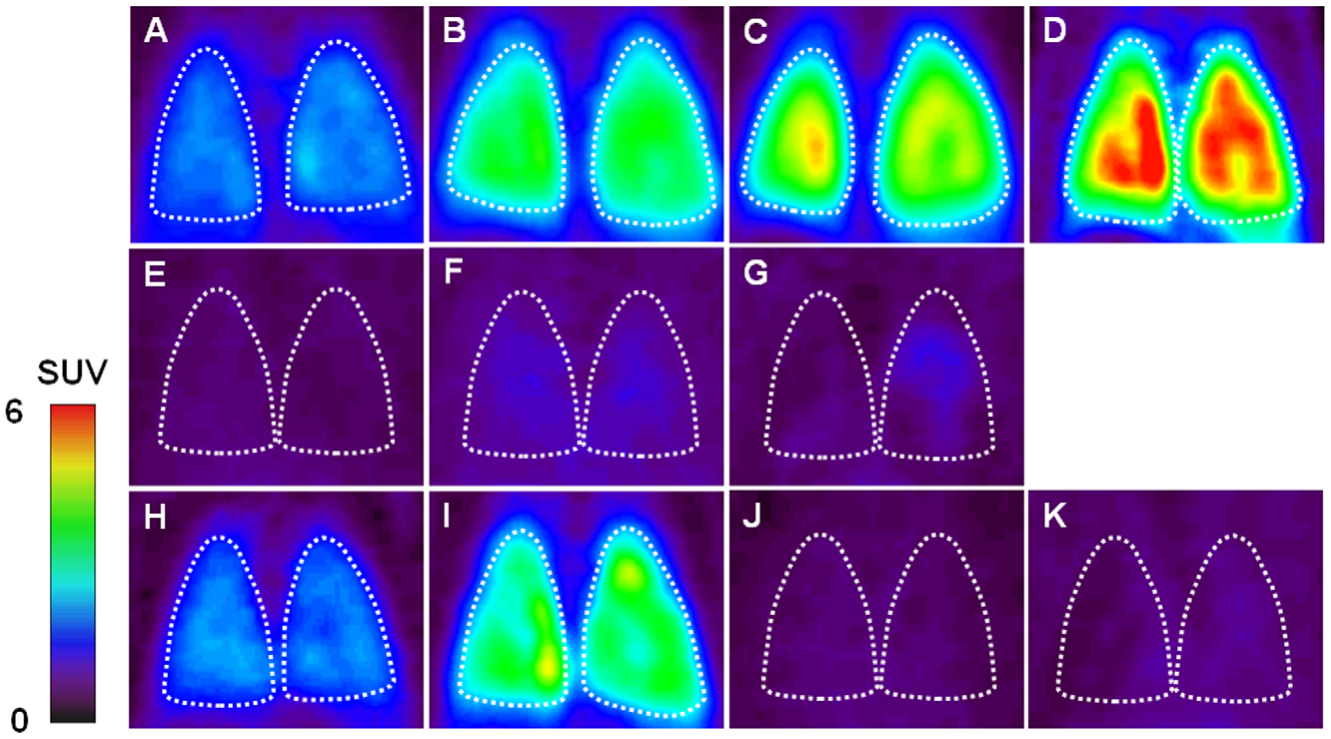
Representative coronal PET lung images acquired between 0 and 30 min after injection of radioligand. [^18^F]FEDAC: (A) control; (B) LPS-2 h; (C) LPS-6 h; (D) LPS-24 h inducement; (E) Pretreatment with PK11195 for control; (F) PK11195 for LPS-6 h; (G) PK11195 for LPS-24 h. [^11^C](*R*)-PK11195: (H) control; (I) LPS-24 h; (J) Pretreatment with PK11195 for control; (K) PK11195 for LPS-24 h. The lung became visible over time after the LPS inducement. Contrast of the [^18^F]FEDAC images between the control and LPS-24 h was greater than that for the [^11^C](*R*)-PK11195 images. Inhibitory experiment with PK11195 diminished the difference in radioactivity among all lung images. [^18^F]FEDAC showed higher lung uptake than [^11^C](*R*)-PK11195.

Figure 1 shows representative PET summation images of rat lungs between 0 and 30 min after injection of [^18^F]FEDAC (A–G) and [^11^C](*R*)-PK11195 (H–K). Compared to the control image of [^18^F]FEDAC (A), radioactivity in the lungs increased 2 h (B), 6 h (C), and 24 h (D) after LPS inducement. The lungs became more visible over time after the inducement. Unlabelled PK11195 (1 mg/kg) was used to examine whether radioactive signal shown in the lung was specific to TSPO. As shown in Fig. 1E–G, PK11195 treatment diminished the difference in radioactivity among all lung images and significantly reduced the lung uptake. On the other hand, compared to the [^18^F]FEDAC image of LPS-24 h (D), the [^11^C]PK11195 image showed weaker signals in the lung at the same time point (I). Contrast of the [^18^F]FEDAC images between the control (A) and LPS-24 h (D) was greater than that for the [^11^C](*R*)-PK11195 images (H, I).

**Figure 2:**
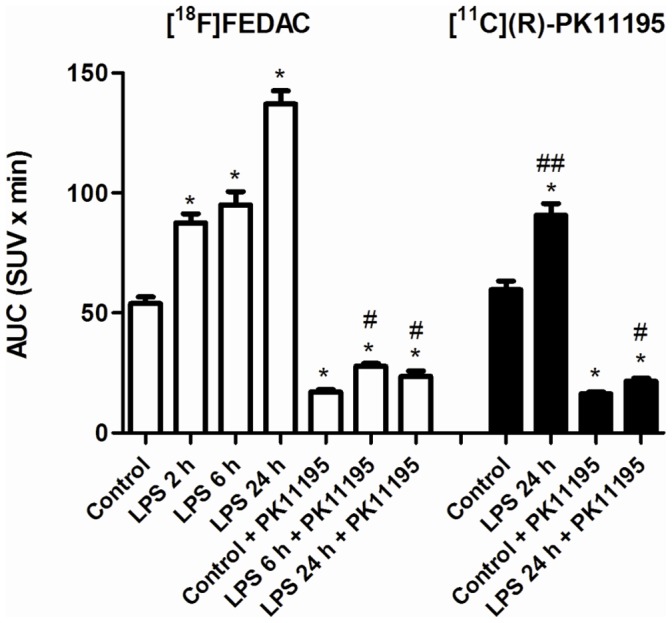
Radioactivity accumulating in the lungs of the control and inflammatory rats. The values of areas under time-activity curves (AUC_0–30 min_; SUV×min, mean ± SEM., *n = *4 for each group) were calculated from the time-activity curves between 0 and 30 min after injection of [^18^F]FEDAC (white bars) and [^11^C](*R*)-PK11195 (black bars). The lung uptake became higher with time after LPS inducement. Unlabelled PK11195 treatment largely reduced the lung uptake. A significant difference (P<0.05) was seen on the following comparisons, *: control vs. LPS-2 h, 6 h, or 24 h, PK11195 treatment for control, LPS-6 h or 24 h; #: LPS-6 h or 24 h vs. PK11195 treatment for LPS-6 h or 24 h; ##: [^18^F]FEDAC vs. [^11^C](*R*)-PK11195 for LPS-24 h.

The uptake of radioactivity in the lungs was quantified based on the time-activity curves (Figure S1 for [^18^F]FEDAC and Figure S2 for [^11^C](*R*)-PK11195) between 0 and 30 min after the radioligand injection. Figure 2 shows the uptake values represented as areas under time-activity curves (AUC_0–30 min_) in the lungs. The uptake of [^18^F]FEDAC accumulating in the lungs increased from 53.9±2.8 of the control to 137.1±5.5 of the LPS-24 h inducement, while the lung uptakes of [^11^C](*R*)-PK11195 in the control and LPS-24 h were 59.7±3.6 and 90.8±4.8, respectively. After treatment with a TSPO-selective ligand PK11195 for the control and LPS-induced rats, the AUC_0–30 min_ values of both radioligands in the lungs were decreased to the similar and low levels.

After the PET scans had finished, all experimental rats were sacrificed and radioactivity levels in the blood and lung were measured. The radioactivity in the blood and lung of control and LPS-treated groups ranged from 0.11 to 0.46 and from 3.66 to 14.57 at 30 min after injection of each radioligand, respectively. By treatment with PK11195, the radioactivity in the blood and lung reduced in ranges of 0.24–1.09 and 0.61–2.65 at 30 min after injection.

### Western Blot Assay

**Figure 3:**
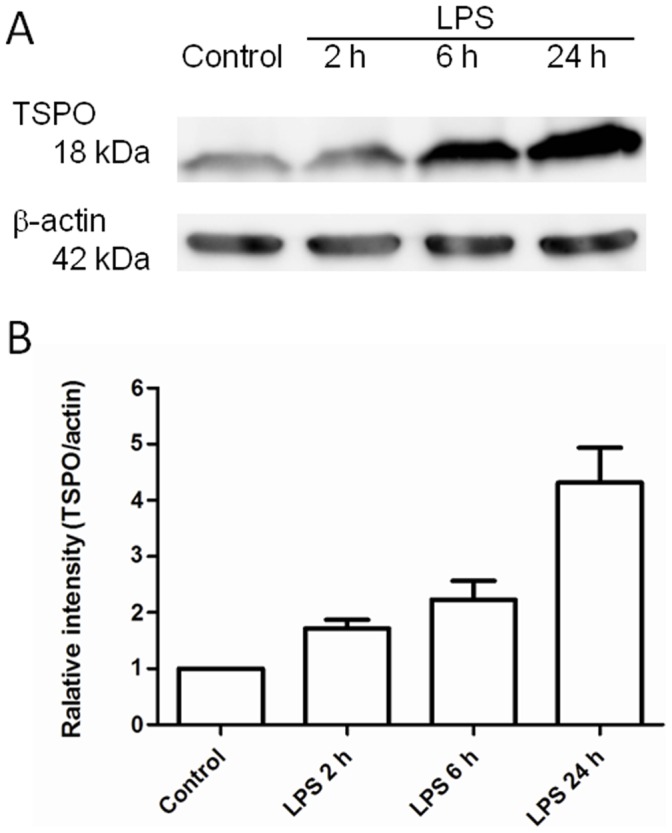
TSPO expression of the control and LPS-induced lungs by Western blot assay. (A) Expression of TSPO and b-actin in lungs. (B) Relative levels of TSPO in the lung sections (*n = *4 for each group). The relative intensity of LPS-2 h, 6 h and 24 h vs. control was compared using each corrected band intensity (TSPO/beta-actin). A low level of TSPO was noted on the band of the control lung. Increase in the TSPO expression was observed over time after LPS inducement. The relative intensities on the bands of LPS-2 h, 6 h, and 24 h, showing a stepwise increase of TSPO expression compared to the control band.

Figure 3 shows the TSPO expression on the lung tissues of the control and LPS-induced rats as determined by Western blot assay. A low level of TSPO was noted on the band of the control lung. Increases in the TSPO expression were observed over time after LPS inducement. The relative intensities on the bands of LPS-2 h, 6 h, and 24 h, showing the degree of TSPO expression compared to the control band, were 1.7, 2.2 and 4.3, respectively.

### Immunohistochemical Staining Assay

**Figure 4:**
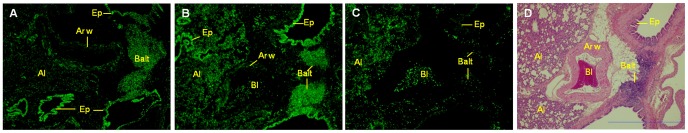
TSPO expression in the lung tissues determined by immunohistochemical assay. (A) TSPO was seen in the intrapulmonary bronchial and bronchiole epithelium, and bronchus-associated lymphoid tissue of the control. (D) Histological alteration was shown on the H&E staining image of the LPS-2 h section. (B, D) TSPO over the alveoli in LPS-2 h increased higher than that in the control, with few over the blood in the artery. (C, D) Many neutrophils over the alveoli and blood in the artery were detected in LPS-2 h. Abbreviations: bronchial and bronchiole epithelium (Ep), bronchus-associated lymphoid tissue (Balt), arterial wall (Ar w), alveoli (Al), blood in artery (Bl). Scale bar: 1 mm.

Figure 4 shows the TSPO expression in the lung tissues of the control (A) and LPS-2 h induced rat (B), and neutrophils on the adjacent section of LPS-2 h (C) determined by immunohistochemical assay. The H&E staining image reflecting the histological alteration on the LPS-2 h section is shown in Fig. 4D. On the control lung, dense fluorescence representing TSPO expression was detected over the intrapulmonary bronchial and bronchiole epithelium, with less fluorescence over bronchus-associated lymphoid tissue (BALT), and with weak signal over the pulmonary arterial wall and alveolar walls (A). Increases in TSPO expression were observed on the LPS-2 h sections (B). TSPO increased on the alveoli of the LPS-2 h section, compared to that of the control. As shown in Fig. 4C, many neutrophils over the alveoli and blood in the artery were detected on LPS-2 h, while few neutrophils were seen on the control section (data not shown).

**Figure 5:**
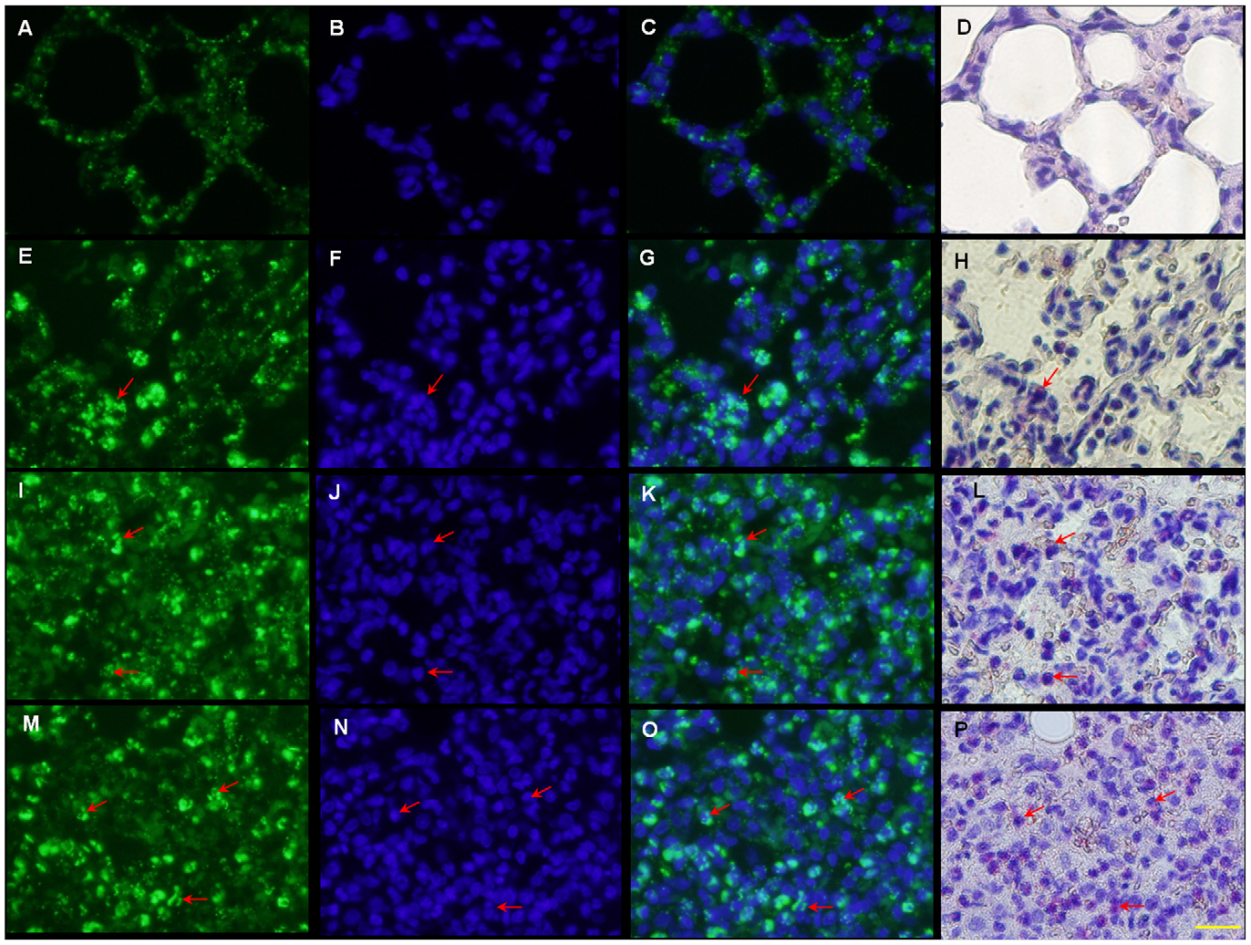
Comparison of the immunofluorescence labeling of TSPO and chloroacetate esterase staining for neutrophils. Arrows indicate examples of cells doubly positive for TSPO (green) and chloroacetate esterase (red spots) staining. (A–D) No neutrophils were seen in the control. (E–H) Many neutrophils showing TSPO immunoreactivity were observed in the alveoli walls of LPS-2 h. (I–L) Some alveoli were collapsed in LPS-6 h, and a considerable number of neutrophils with TSPO infiltrated into the alveolar spaces. (M–P) Further alveoli collapse and neutrophil infiltration were seen in LPS-24 h. Scale bar: 20 µm.

**Figure 6:**
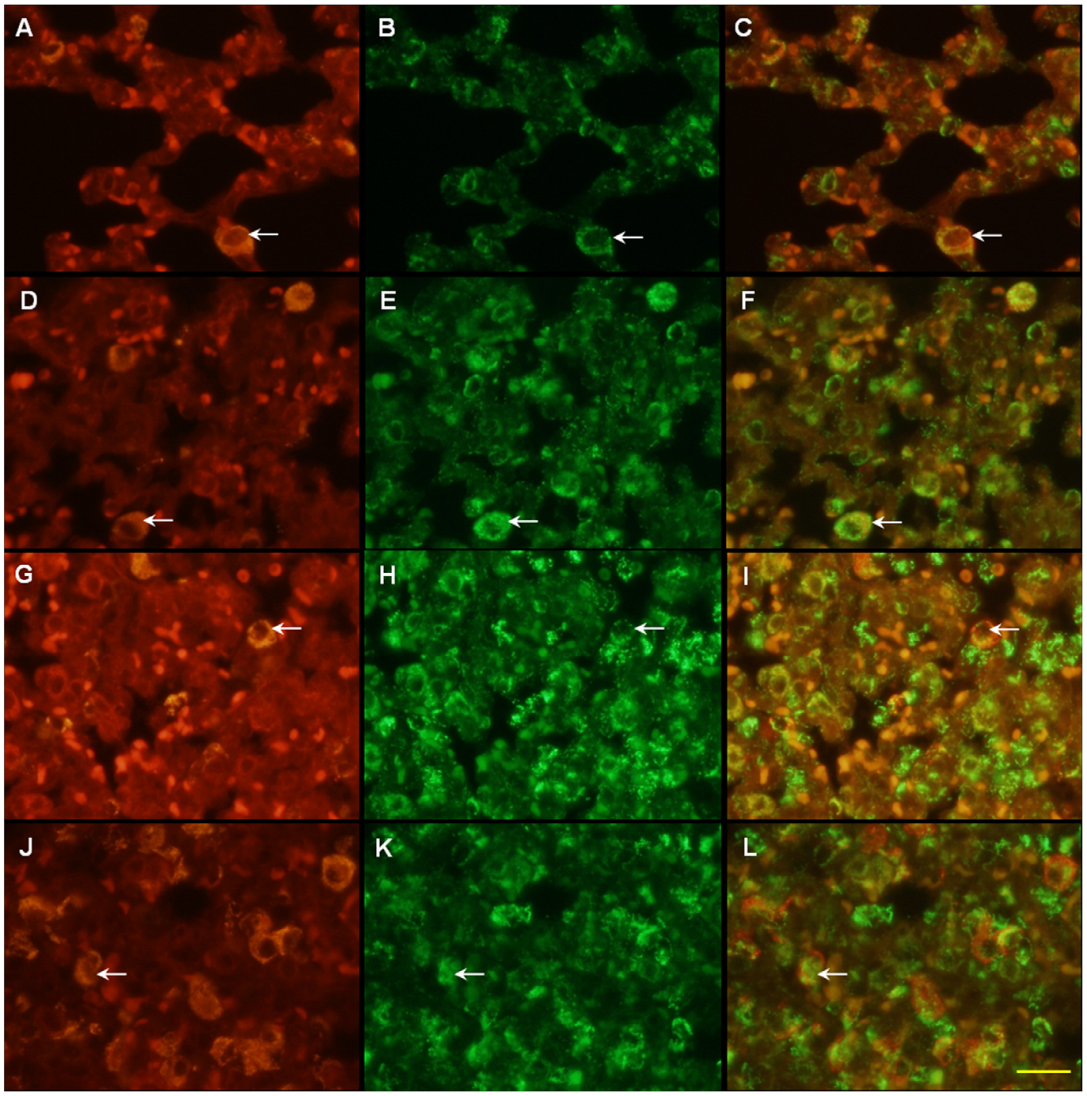
Double immunofluorescence labeling of ED1 (red) and TSPO (green) displayed as a two-channel image. The images demonstrated temporal alterations of TSPO expression in macrophages at 2 h (D–F), 6 h (G–I) and 24 h (J–L) after LPS inducement, and in the control (A–C). Arrows indicate examples of cells doubly positive for ED1 and TSPO. (A–C) TSPO expression was seen in a few macrophages. (D–F) Several macrophages showing TSPO immunoreactivity were observed in the alveolar spaces. (G–I, J–L) Numbers of macrophages with TSPO increased gradually over time. Scale bar: 20 µm.

Because alveoli were affected remarkably by the intratracheal administration of LPS, double staining of TSPO was performed for neutrophils (Fig. 5) and macrophages (Fig. 6) on the alveoli, respectively.

On the control lung sections, neutrophils were not observed in the alveolar part (Fig. 5A–D) and TSPO was shown in a few macrophages (Fig. 6A–C). There were many deformed alveolar spaces on the LPS-2 h sections (Fig. 5E–H, 6D–F). Many neutrophils in the alveoli walls and several macrophages in the alveolar spaces showed TSPO immunoreactivity. In the LPS-6 h sections, some of the alveoli were collapsed and a considerable number of neutrophils with TSPO had infiltrated into the alveolar spaces (Fig. 5I–L). On the LPS-24 h sections, further alveoli collapse and neutrophil infiltration were observed (Fig. 5M–P). After the LPS inducement, macrophages increased gradually until 24 h while neutrophils increased immediately and rapidly from the start of inducement (Fig. 6D–L). The numbers were 7.0±0.9, 54.0±1.6, 157.1±12.2 and 162.8±10.2 for TSPO-positive neutrophils and 6.7±0.7, 14.8±1.6, 31.0±6.5, and 45.6±4.5 for TSPO-positive macrophages on 0.035 mm^2^ of the control, LPS-2 h, 6 h, and 24 h induced lung sections, respectively. The present results suggest that neutrophils preferably contributed to the development of TSPO expression until 24 h after LPS inducement in this model.

## Discussion

In the present study, PET with [^18^F]FEDAC can visualize lung inflammation in the rat model of acute lung injury. Western blot assay indicated that increases of TSPO expression corresponded to increases of [^18^F]FEDAC uptake in the lung. Significant elevation of TSPO level was confirmed in the inflamed lung sections. The [^18^F]FEDAC uptake specific to TSPO increased with the activation of neutrophils and macrophages in the lung.

[^18^F]FEDAC is a useful PET ligand for the imaging of TSPO developed in our group [18]–[20]. PET with [^18^F]FEDAC in normal rats has shown high in vivo specific binding for TSPO and high radioactive signals in the TSPO-enriching adrenal gland, lung, heart, and kidney [19]. On the other hand, the uptakes of [^18^F]FEDAC in liver and blood were much lower than that in lung, showing that the imaging of peripheral organs could not disturbed by the liver and blood uptakes. Moreover, in vivo metabolite analysis showed that [^18^F]FEDAC was stable in the lung, heart, and kidney. This profile guaranteed that [^18^F]FEDAC has good potential as an imaging agent for peripheral organs or tissues.

There is experimental evidence that LPS induces intense lung inflammation, with macrophage activation and recruitment of neutrophils to the interstitium, alveoli, and airways of rats [21]–[25]. Moreover, inappropriate activity of neutrophil and macrophage is known to be involved in a number of common lung diseases. We thus used LPS to prepare a rat model of acute lung injury and validated the usefulness of PET with [^18^F]FEDAC for the imaging of lung inflammation. Because of technical problem, we could not use the same rats as controls (i.e. induced LPS on left lung, and use the right side of the lung as control).

In the present study, we performed small-animal PET scans with [^18^F]FEDAC for the rats at 2 h, 6 h, and 24 h after LPS inducement to monitor the progress of lung inflammation (Fig. 1, 2). A comparison of lung images and AUC_0–30 min_ values between the control and inflammation groups indicated elevated levels of radioactivity retention in the inflamed lungs. The PET images showed an increase of uptake and radioligand binding in the lungs with the progress of lung inflammation. At 24 h after inducement, the AUC_0–30 min_ value of [^18^F]FEDAC increased to 2.5 fold, compared to that of the control. Treatment with the TSPO-specific ligand PK11195 significantly reduced lung uptakes and abolished radioactive signals on the PET images. These results demonstrated that the uptake and binding of [^18^F]FEDAC in the lungs was specific to TSPO. At 30 min after injection of each radioligand, the uptake ratio of lung to blood was higher than 13 for the rats without PK11195 treatment, whereas the corresponding ratio was 2–3 for the PK11195-treated rats. Although quantitative analysis of TSPO binding might be affected by the blood uptake to some extent, qualitative analysis might not. These results indicate that the present lung images are not confounded by the blood uptakes of both radioligands.

The presence of TSPO in the lung was confirmed using a Western blot assay. In comparison to the control, TSPO was detected in the inflamed lungs at higher levels (Fig. 3). TSPO expression increased with the severity of inflammation which was caused by prolonging the time after LPS inducement. This finding indicated that the elevated TSPO level and radioactivity may reflect the progress of lung inflammation.

The cellular sources of TSPO levels were assessed in the lung tissues. There have been reports that TSPO is expressed in circulating blood cells with high concentrations in polymorphonuclear neutrophils and monocytes [29], [30]. TSPO was also found to express in Schwann cells and macrophages of the peripheral nervous system, with increased expression in animal models of peripheral nerve injury and macrophage activation [31]. In this present study, TSPO expression was recognized over bronchial and bronchiole epithelium, BALT and alveolar walls of LPS-induced rat lung (Fig. 4). Immunohistological analysis demonstrated that TSPO was highly expressed in the activated neutrophils and macrophages by LPS inducement. After the inducement, neutrophils immediately increased at a rapid speed while macrophages increased gradually until 24 h. This finding suggests that the binding sites of [^18^F]FEDAC for TSPO are numerous not only on macrophages but also on neutrophils in the present lung inflammation. It should be noted that although the numbers of neutrophils and macrophages increased with uptakes of [^18^F]FEDAC in lung sections over time, increase of TSPO expression in the lung was due to the cell activation but was not proportional to the cell numbers.

In addition to neutrophils and macrophages, lymphocytes may also be the cellular sources that are expressing TSPO. In fact, TSPO expression was observed in BALT of the control and LPS-induced lungs by the immunohistochemical assay. Because BALT is a lymphoid aggregate containing both B and T lymphocytes and is located in the submucosal area of the bronchi and bronchioles, BALT is known to play a central role in airway mucosal immunity by inducing of secretory IgA [32]. TSPO expression in B and T lymphocytes may also be partly responsible for the increase in the [^18^F]FEDAC uptake in the inflamed lung.

[^11^C](*R*)-PK11195 as a standard PET ligand for TSPO has been previously used for the imaging of lung inflammation [11]–[13]. In our experiment using the rat model of acute lung injury, the AUC_0–30 min_ value of [^11^C](*R*)-PK11195 in the LPS-24 h lung was lower than that of [^18^F]FEDAC (Fig. 2). Difference between the control and inflammation groups for [^11^C](*R*)-PK11195 were also smaller than that for [^18^F]FEDAC. Moreover, the specific binding, which was determined by subtracting the binding after PK11195-treatment from the total binding, was higher for [^18^F]FEDAC than [^11^C](*R*)-PK11195. This comparison suggested a higher sensitivity of [^18^F]FEDAC for the imaging of lung inflammation than that of [^11^C](*R*)-PK11195. Also, [^18^F]FEDAC is more convenient and useful than [^11^C](*R*)-PK11195 because it can be delivered to other facilities without a cyclotron owing to a longer half-life of ^18^F (110 min) than that of ^11^C (20 min).

It has been reported that PET with [^11^C](*R*)-PK11195 can assess the macrophage activation of COPD patients [12]. In a rabbit model following a particulate challenge, PET images at 3 and 6 days after the challenge indicated that macrophages, but no neutrophils, contributed to the signal of [^11^C](*R*)-PK11195 [11], [13]. Our present results indicated that both neutrophils and macrophages mainly contributed to the [^18^F]FEDAC signals in the lung. The difference between previous studies and our findings may be due to the progressing period after the inducement of inflammation. Our present model reflected an early phase of lung inflammation, in which macrophage activation was slow, while neutrophils were activated rapidly and largely after LPS inducement. There were also reports that phagocytosis of macrophages was less active than that of neutrophils in the early phase of acute lung injury, although macrophages are known to play an important role as a trigger of the acute injury [33], [34]. Thus, neutrophils in the early inflammation may be contributing to the increase of TSPO expression and [^18^F]FEDAC uptake more significantly than macrophages.

The present findings suggest the possibility of a PET-TSPO study. PET with [^18^F]FEDAC may be useful for monitoring activation of macrophages as well as neutrophils in the early lung inflammation. For clinic usefulness, early evaluation of acute lung injury and acute respiratory distress syndrome is important for timely and accurate therapeutic intervention for patients suffering from these diseases. On the other hand, [^18^F]FDG is also available for the in vivo assessment of lung inflammation [7]–[10]. However, [^18^F]FDG is not only a radioligand for inflammation, as it is mostly used for the imaging of various tumors [35]. Although [^18^F]FDG-PET can evaluate glucose metabolism of neutrophils in lung, [^18^F]FEDAC is more specific than [^18^F]FDG because TSPO is a specific marker that is increased in the inflammatory process. Therefore, human study on lung inflammation using PET with [^18^F]FEDAC is promising.

### Conclusion

[^18^F]FEDAC-PET is a useful tool for imaging TSPO and monitoring the progress of lung inflammation. The present study will contribute to the determination of the pathogenic progress and will be of use in evaluating the therapeutic effects of anti-inflammatory drugs. In this future validation study, we hope to determine if the radioactive signal of [^18^F]FEDAC is sufficiently high to enable detection within the inflamed lungs of human subjects.

## Supporting Information

Figure S1 **Time-activity curves in lungs of control and LPS-induced rats after intravenous injection of [^18^F]FEDAC.** The uptake of radioactivity in the lungs increased 2 h, 6 h, and 24 h after LPS inducement compared to the control. Pretreatment with PK11195 significantly reduced the uptake of radioactivity in lungs.
(DOC)

Figure S2 **Time-activity curves in lungs of control and LPS-24 h induced rats after intravenous injection of [^11^C](** ***R*** **)-PK11195.** The radioactivity in the lungs increased LPS-24 h inducement compared to the control. Pretreatment with PK11195 significantly reduced the uptake of radioactivity in lungs. Differences between the control and LPS-24 h inducement for [^11^C](*R*)-PK11195 were smaller than those for [^18^F]FEDAC.
(DOC)
