# Supplementary material for: PET Imaging of Lung Inflammation with [18F]FEDAC, a Radioligand for Translocator Protein (18 kDa)
Source: PLoS One. 2012 Sep 12;7(9):e45065. doi: 10.1371/journal.pone.0045065 (PMC3440397; doi:10.1371/journal.pone.0045065)
Supplement: Figure S1 — Time-activity curves in lungs of control and LPS-induced rats after intravenous injection of [18F]FEDAC. The uptake of radioactivity in the lungs increased 2 h, 6 h, and 24 h after LPS inducement compared to the control. Pretreatment with PK11195 significantly reduced the uptake of radioactivity in lungs. (DOC) [file pone.0045065.s001.doc]

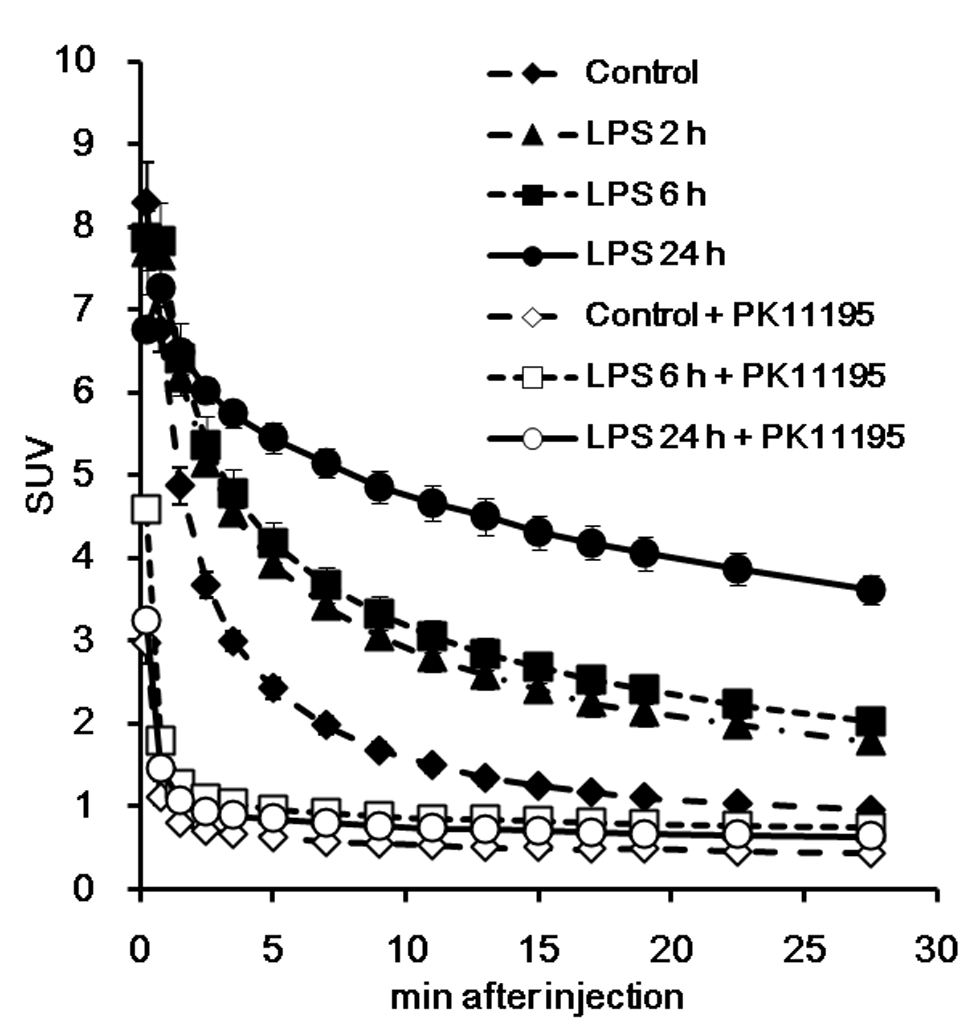


**Supporting Figure 1**. **Time-activity curves in lungs of control and LPS-induced rats after intravenous injection of [^18^F]FEDAC.**

The uptake of radioactivity in the lungs increased 2 h, 6 h, and 24 h after LPS inducement compared to the control. Pretreatment with PK11195 significantly reduced the uptake of radioactivity in lungs.
