# Supplementary material for: PET Imaging of Lung Inflammation with [18F]FEDAC, a Radioligand for Translocator Protein (18 kDa)
Source: PLoS One. 2012 Sep 12;7(9):e45065. doi: 10.1371/journal.pone.0045065 (PMC3440397; doi:10.1371/journal.pone.0045065)
Supplement: Figure S2 — Time-activity curves in lungs of control and LPS-24 h induced rats after intravenous injection of [11C]( R )-PK11195. The radioactivity in the lungs increased LPS-24 h inducement compared to the control. Pretreatment with PK11195 significantly reduced the uptake of radioactivity in lungs. Differences between the control and LPS-24 h inducement for [11C](R)-PK11195 were smaller than those for [18F]FEDAC. (DOC) [file pone.0045065.s002.doc]

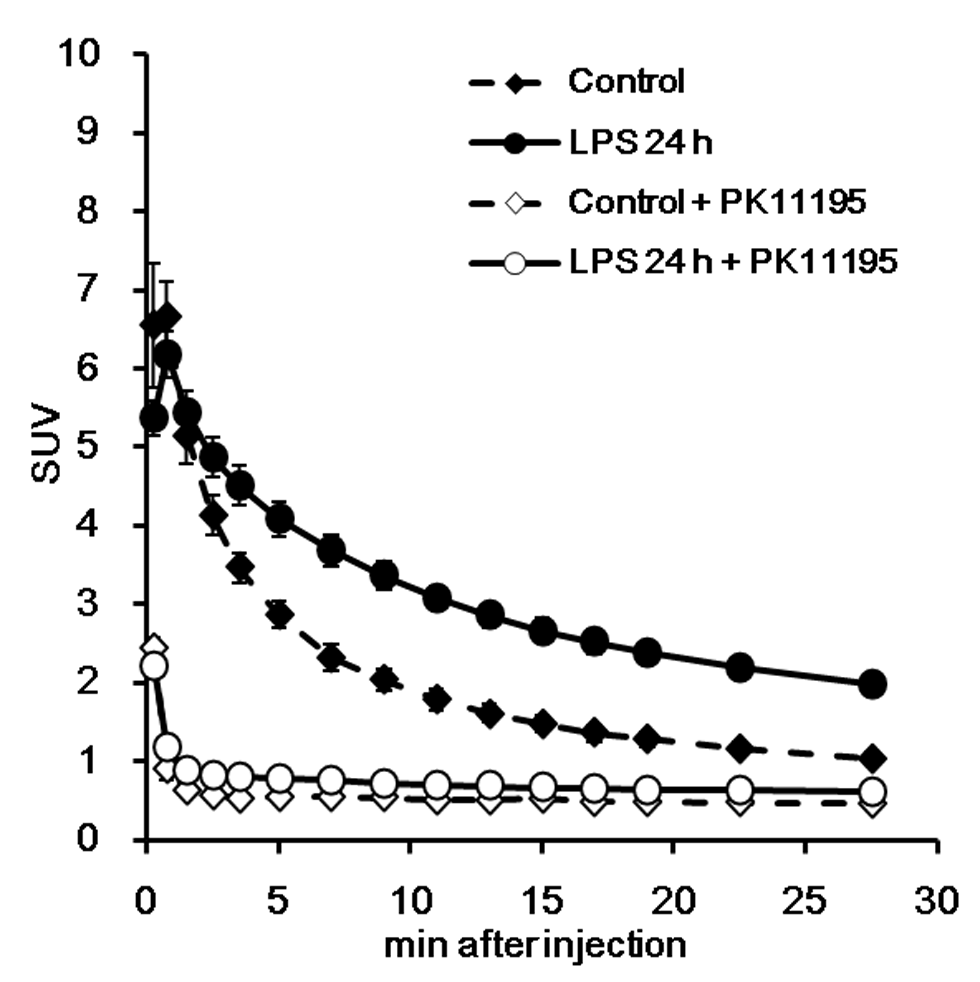


**Supporting Figure 2**. **Time-activity curves in lungs of control and LPS-24 h induced rats after intravenous injection of [^11^C](*R*)-PK11195**.

The radioactivity in the lungs increased LPS-24 h inducement compared to the control. Pretreatment with PK11195 significantly reduced the uptake of radioactivity in lungs. Differences between the control and LPS-24 h inducement for [^11^C](*R*)-PK11195 were smaller than those for [^18^F]FEDAC.
